# Disrupted stepwise functional brain organization in overweight individuals

**DOI:** 10.1038/s42003-021-02957-7

**Published:** 2022-01-10

**Authors:** Hyebin Lee, Junmo Kwon, Jong-eun Lee, Bo-yong Park, Hyunjin Park

**Affiliations:** 1 Department of Electrical and Computer Engineering, Sungkyunkwan University, Suwon, Korea; 2 Center for Neuroscience Imaging Research, Institute for Basic Science, Suwon, Korea; 3 Department of Data Science, Inha University, Incheon, Korea; 4 School of Electronic and Electrical Engineering, Sungkyunkwan University, Suwon, Korea

**Keywords:** Image processing, Network models

## Abstract

Functional hierarchy establishes core axes of the brain, and overweight individuals show alterations in the networks anchored on these axes, particularly in those involved in sensory and cognitive control systems. However, quantitative assessments of hierarchical brain organization in overweight individuals are lacking. Capitalizing stepwise functional connectivity analysis, we assess altered functional connectivity in overweight individuals relative to healthy weight controls along the brain hierarchy. Seeding from the brain regions associated with obesity phenotypes, we conduct stepwise connectivity analysis at different step distances and compare functional degrees between the groups. We find strong functional connectivity in the somatomotor and prefrontal cortices in both groups, and both converge to transmodal systems, including frontoparietal and default-mode networks, as the number of steps increased. Conversely, compared with the healthy weight group, overweight individuals show a marked decrease in functional degree in somatosensory and attention networks across the steps, whereas visual and limbic networks show an increasing trend. Associating functional degree with eating behaviors, we observe negative associations between functional degrees in sensory networks and hunger and disinhibition-related behaviors. Our findings suggest that overweight individuals show disrupted functional network organization along the hierarchical axis of the brain and these results provide insights for behavioral associations.

## Abstract (web summary)

Hyebin Lee et al. use a stepwise functional connectivity technique to study differences in functional connectivity between overweight and healthy weight individuals. They find disrupted functional network organization along the hierarchical axis of the brain in overweight individuals that relates to behavioral traits

## Introduction

Obesity is a state of high body mass index (BMI) and is recognized as a risk factor for severe health problems, such as type 2 diabetes, cardiovascular diseases, stroke, and various cancers^1–4^. Multiple neurobiological studies for individuals with obesity reported associations between disrupted reward/executive control systems and behavioral traits, as well as genetic underpinnings^5–11^, motivating neuroimaging investigation of obesity.

Magnetic resonance imaging (MRI) is a powerful neuroimaging technique evaluating brain structure and function in vivo. It has been widely used for assessing whole-brain morphology and functional response, as well as connectivity, and resulted in various correlates of obesity^12–19^. Previous studies observed that individuals with obesity showed differences in gray matter volume in the sensorimotor and transmodal regions of frontal and temporal cortices^19^, as well as decreases in cortical thickness in reward systems, including the orbitofrontal cortex, ventral diencephalon, and brainstem^14^. In addition to morphological alterations, functional connectivity perturbation, particularly in the regions related to reward systems, has been observed in previous neuroimaging studies based on resting-state functional MRI (rs-fMRI)^15–17^. Task-based fMRI studies have shown enhanced food-related responses during reward processing in reward and default-mode networks^12,13^. Recent studies from our group and others combined functional connectivity analysis with machine learning and provided whole-brain connectome signatures associated with obesity phenotypes, suggesting notable connectional alterations in sensory, and transmodal areas^20,21^. We previously showed that higher BMI is associated with an elevated level of functional connectivity in executive control and reward systems, whereas sensory regions showed decreased sensitivity^21,22^. In regards to the graph-theoretical parameters, increased segregation of modular architecture was observed in heteromodal association cortices^23,24^. In summary, obesity may be characterized by alterations in hierarchical functional brain organization, spanning from sensory to default-mode and control networks.

The cortical hierarchy suggested by Mesulam was formulated in non-human primates, and it involves the following four levels of neural organization: idiotypic, unimodal association, heteromodal association, and paralimbic cortices^25^. This concept has been expanded to human models, suggesting a hierarchical axis differentiating sensorimotor regions from a transmodal anchor^26^. Functional brain hierarchy can be effectively evaluated using a technique called stepwise functional connectivity (SFC)^27^. SFC is an expansion of the conventional seed-based functional connectivity approach, and this analysis counts the number of all possible paths that connect different brain regions with specific step distances^27^. The approach evaluates direct connections to indirect connections involving a varying number of step distances. Thus, it is suitable to characterize gradual changes in functional connectivity from the primary sensory to association cortices^27^. SFC analysis has been adopted in previous studies to assess developmental changes in the hierarchical organization of the brain^28^, as well as to estimate perturbed functional connectivity in diseased populations with attention-deficit/hyperactivity disorder and autism spectrum disorder^29–31^. As overweight individuals show disrupted hierarchical organization in brain function^20–22,24^, the SFC analysis would be an appropriate approach for investigating the perturbation of functional brain hierarchy in overweight individuals. We thus hypothesized that the hierarchical organization of functional brain networks may show alterations in overweight individuals relative to healthy weight controls.

In this study, we investigated perturbations of functional connectivity across different step distances in individuals with overweight. First, we defined seed regions for SFC analysis by associating obesity phenotypes with graph-theoretical measures previously established in obesity-related studies to consider the continuous nature of obesity phenotypes, such as BMI and waist-to-hip ratio (WHR). We then assessed distinct patterns of functional connectivity between individuals with healthy weight and overweight at different step distances to assess perturbations of functional hierarchy in the overweight group. Finally, we associated functional connectivity with eating behaviors to examine underlying behavioral traits.

## Results

**Fig. 1:**
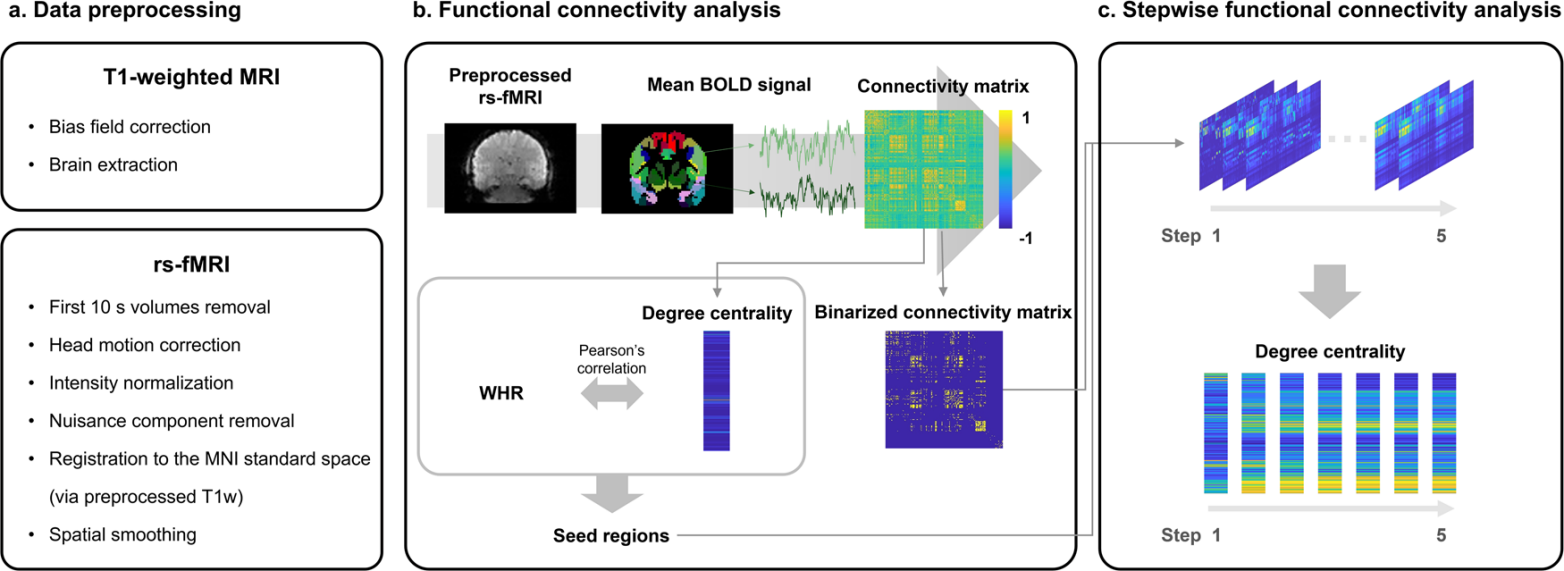
Flowchart of the study. **a** The T1-weighted MRI and rs-fMRI data were preprocessed. **b** (top) We calculated partial correlations of mean time series between different brain regions defined using Brainnetome atlas, (bottom) and calculated degree centrality to assess association to waist-to-hip ratio (WHR). The regions that showed significant associations were selected as seed regions for stepwise functional connectivity (SFC) analysis. **c** (top) The SFC analysis was performed using binarized connectivity matrix from steps one to five, and (bottom) degree centrality was calculated for each step. *Abbreviations:* rs-fMRI resting-state functional magnetic resonance imaging, BOLD blood-oxygen-level-dependent.

We studied 301 participants (mean ± standard deviation age = 40.44 ± 17.68 years; 60.47% female) with a wide range of BMI (16.25~47.49) and WHR (0.59~1.18) obtained from the enhanced Nathan Kline Institute-Rockland Sample (eNKI) database^32^. Details on participant demographics, image acquisition and processing, and SFC analysis can be found in *Methods*, and overall flow is described in Fig. 1.

### Seed regions associated with obesity phenotype

**Fig. 2:**
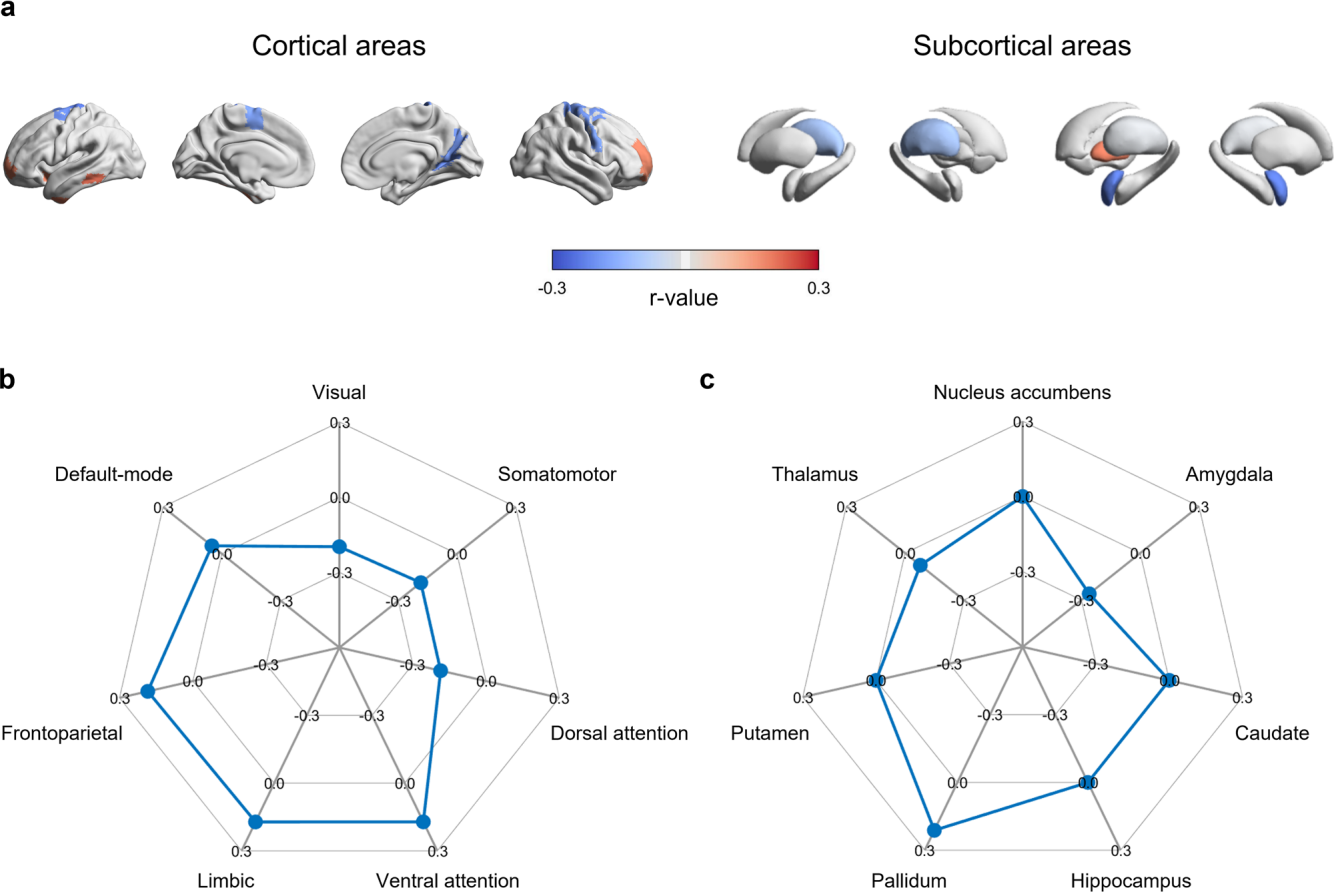
Brain regions associated with obesity phenotype. **a** Correlation coefficient of the identified regions that showed significant association with waist-to-hip ratio (WHR) are reported on brain surfaces and subcortical structures using 301 participants. Red/blue indicate positive/negative correlations. The correlation coefficients are stratified based on **b** functional communities as well as **c** subcortical regions.

We constructed functional connectivity matrix from rs-fMRI data of all participants and calculated degree centrality values (Fig. 1a, b; see *Methods*). We linearly correlated the degree centrality with WHR to define seed regions for SFC analysis, and 26 brain regions showed significant associations (*q* < 0.05; Fig. 2a). Specifically, ventrolateral prefrontal, inferior temporal, insular cortices, and pallidum were positively associated with WHR, whereas sensorimotor, visual, thalamus, and amygdala showed negative associations. Stratifying the correlation coefficients based on functional communities^33^, the frontoparietal network showed the highest positive effect, followed by limbic and ventral attention networks, and negative effects were observed in sensory and dorsal attention networks (Fig. 2b). Similarly, the pallidum showed a strong positive association with WHR, and the amygdala and thalamus showed negative associations (Fig. 2c). Based on the previously provided functional profiles of subregions in the Brainnetome atlas^34^, we could find that eleven regions were involved in the cognition, four reward, ten sensorimotor, and one emotion-related functions. In brief, these profiles were determined by forward and reverse inferences, which decode behavioral domains and paradigms based on the BrainMap database (http://www.brainmap.org/taxonomy). Across 1,000 bootstraps, we observed largely consistent seed regions (mean ± standard deviation r = 0.85 ± 0.05; Supplementary Fig. 1), ensuring the robustness (see *Methods*). In the case of BMI, instead of WHR, we observed a consistent albeit lower effect of spatial associations with degree centrality (Supplementary Fig. 2). Indeed, linear correlation between the effects of BMI and WHR was significant (*r* = 0.51, *p* < 0.001).

### Between-group differences in stepwise functional connectivity

**Fig. 3:**
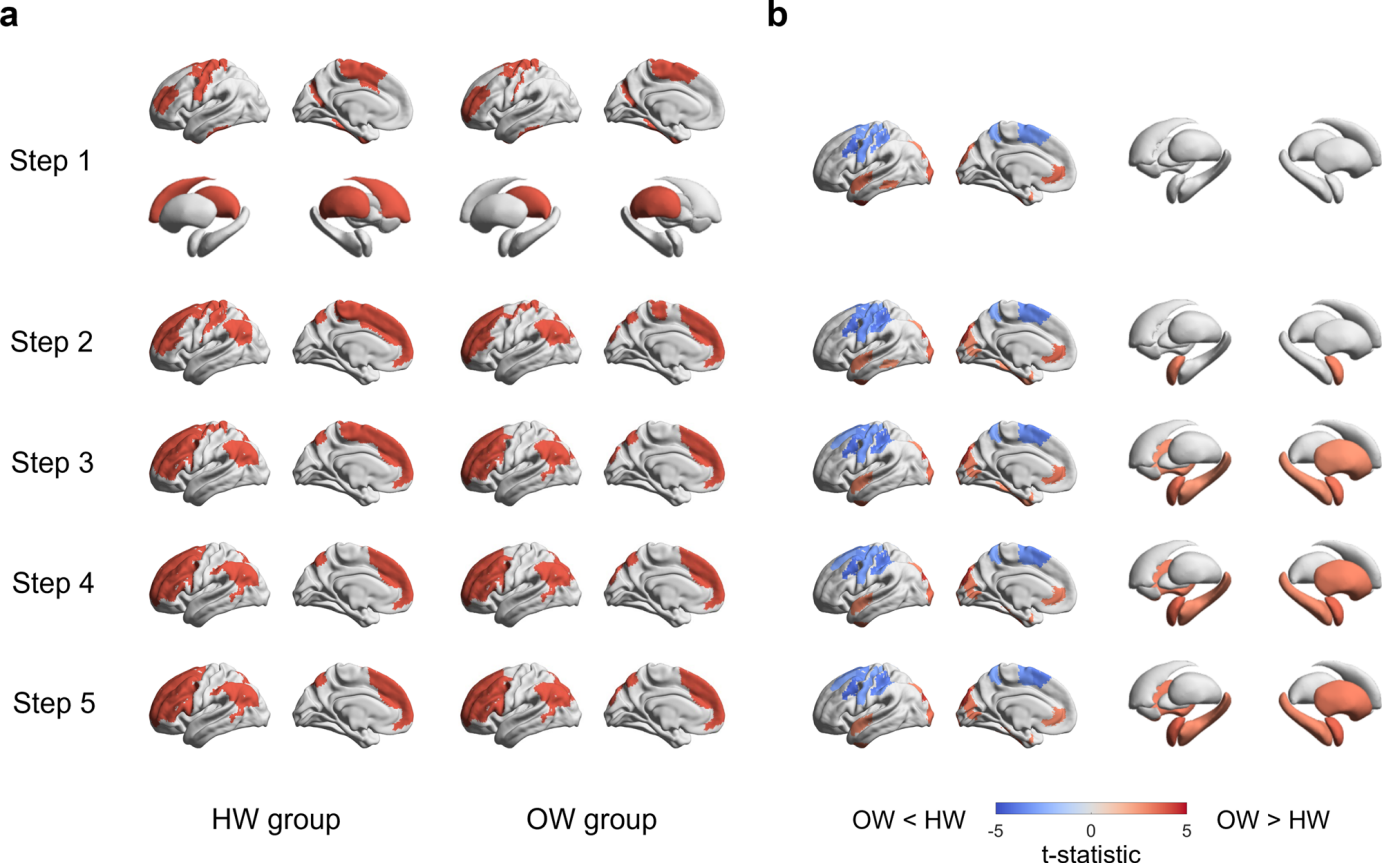
Stepwise functional connectivity in healthy weight (HW) and overweight (OW) groups. **a** Hub regions from step distance one to five for each group (*n* = 104 for HW; *n* = 75 for OW) are reported on brain surfaces. Hub regions in subcortical areas were detected only in the first step. **b** We reported the t-statistics of brain regions that showed significant between-group differences in degree centrality between individuals with HW and OW. Regions with red show a higher degree in OW group compared to individuals with HW, and blue regions, vice versa.

By utilizing the SFC analysis seeded from the identified regions significantly associated with obesity phenotype, we assessed changes in degree centrality at different steps for each group. We specifically tracked hub regions with high degrees, where the normalized degree was higher than 1.5 times the mean degree centrality^35^. Although both groups showed high degree centrality values in the sensorimotor and frontal regions as well as the caudate and thalamus in the first step (equivalent to conventional functional connectivity), stronger connections were observed in the frontoparietal and default-mode networks in higher steps (Fig. 3a). Noting that the connections did not change largely above the step distance five (Supplementary Fig. 3), we reported the results from steps one to five.

**Fig. 4:**
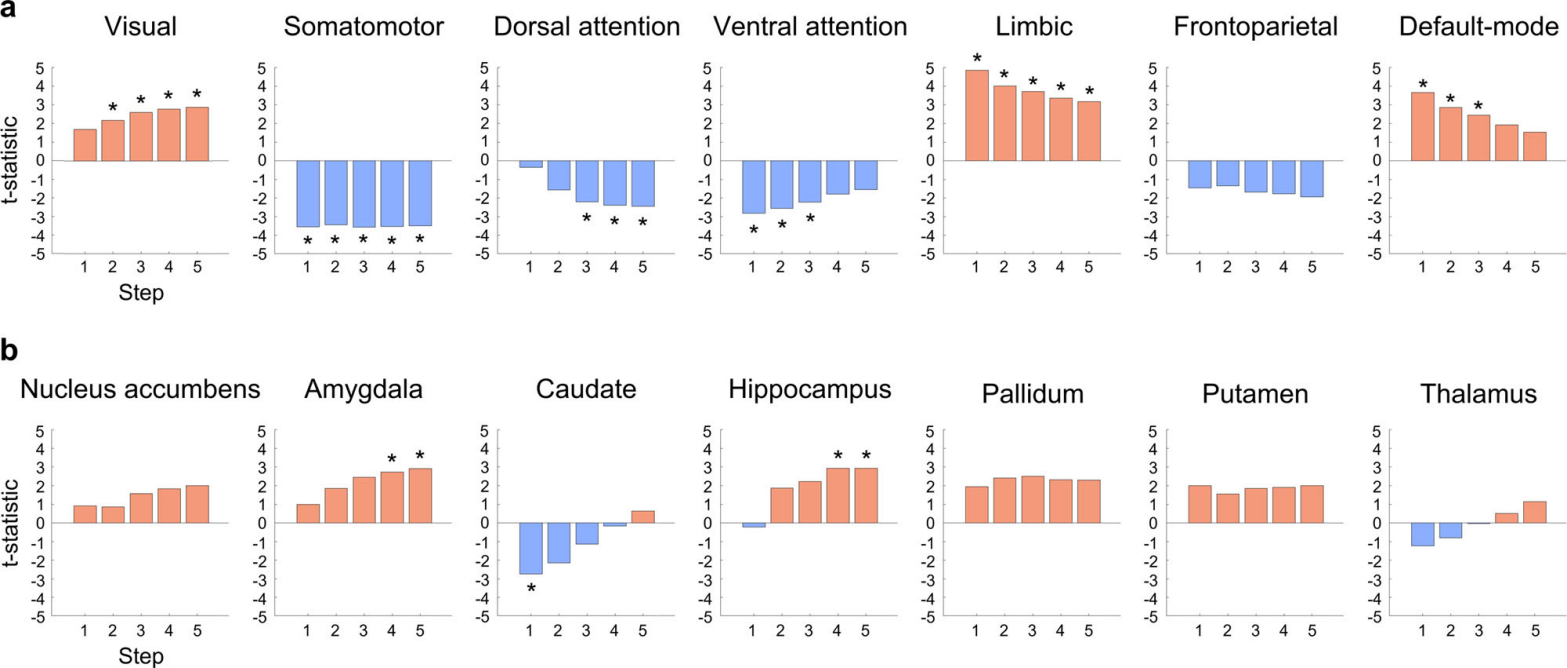
Between-group differences in degree centrality values. We stratified effects of between-group differences in degree centrality values according to **a** functional communities and **b** subcortical regions. Positive (red) values indicate higher degrees in OW group, whereas negative (blue) values indicate lower degrees. The t-statistics with significant between-group differences are marked with asterisks.

We then compared the degree centrality values between individuals with healthy weight and overweight for each step. In the first step, the overweight group showed a higher degree in the medial frontal, lateral temporal, and visual cortices, and a lower degree in sensorimotor regions relative to the healthy weight group (Fig. 3b). The between-group differences were more marked at higher step distances, and we could additionally find higher degrees in the amygdala, hippocampus, putamen, and pallidum. Stratifying the effects based on functional communities^33^, visual, limbic, and default-mode regions showed a higher degree in the overweight group, and a lower degree in the somatomotor, dorsal attention, and ventral attention networks (Fig. 4a). In subcortical regions, the amygdala and hippocampus showed higher degrees in the overweight group, while caudate showed a lower degree only in the first step (Fig. 4b). To assess robustness, we repeated the SFC analysis with different subsets of participants and observed virtually identical patterns of degree centrality values across different step distances (Supplementary Fig. 4; see Methods). We performed the same analyses on subnetworks of (i) cognition, (ii) reward, and (iii) sensorimotor-related regions and found virtually identical results (Supplementary Figs. 5–7).

### Association with eating behaviors

To explore possible underlying behavioral traits of our findings, we associated TFEQ scores with degree centrality values of visual, somatomotor, dorsal attention, and limbic networks, as well as subcortical regions that showed significant between-group differences at a step distance of five. We observed significant negative associations between TFEQ-hunger and visual network (*r* = −0.18, *q* = 0.020), somatomotor network and TFEQ-disinhibition (*r* = −0.17, *q* = 0.025), total without (*r* = −0.17, *q* = 0.03), and with hunger subscale (*r* = −0.15, *q* = 0.043; Table 1).

**Table 1:** Correlations between network-level degree centrality values at step distance five and eating behaviors.

Network	Score	Dietary restraint (1)	Disinhibition (2)	Hunger (3)	(1 + 2)	Total (1 + 2 + 3)
Visual	*r*-value	0.0262	−0.0623	−0.1840*	−0.0134	−0.0839
	*q*-value	0.8155	0.5007	0.0200*	0.9113	0.3580
Somatomotor	*r*-value	−0.1147	−0.1665*	−0.0488	−0.1704*	−0.1506*
	*q*-value	0.2360	0.0250*	0.5639	0.0300*	0.0425*
Dorsal attention	*r*-value	−0.0880	−0.0111	−0.0009	−0.0699	−0.0542
	*q*-value	0.3580	0.9113	0.9880	0.4467	0.5231
Limbic	*r*-value	0.0727	0.0193	0.0060	0.0630	0.0509
	*q*-value	0.4467	0.8438	0.9379	0.5007	0.5429
Subcortical	*r*-value	0.1070	0.0332	0.0537	0.0953	0.0948
	*q*-value	0.2917	0.7111	0.5231	0.3388	0.3388

The *r*- and false discovery rate (FDR)-corrected *p*-values (*q*-values) are reported. Significant results are reported with asterisks.

### Results of sensitivity analysis

- Age and sex. When we controlled age and sex from the functional connectivity, we observed consistent results (Supplementary Fig. 8).
- TFEQ scores. When controlling for eating behavior scores, we could observe consistent SFC patterns (Supplementary Fig. 9), indicating robustness.
- Low- vs. high-health risk groups comparison. Comparing SFC patterns between low- and high-health risk groups defined using the world health organization (WHO) criteria based on WHR^36^, we observed virtually identical results (Supplementary Fig. 10), indicating consistency of our results compared to the between-group differences between overweight and healthy weight groups defined based on both BMI and WHR.

## Discussion

Obesity is a trait affecting brain function, particularly sensory and executive control processing, which are at the opposite ends of the hierarchical spectrum. In this study, we investigated how functional network organization changes at different step distances between individuals with healthy weight and overweight. By leveraging the SFC framework, we found that the step distance-related functional connectivity of the two groups converged in different ways. Although the hub regions of both groups were transmitted from sensory/frontal regions to frontoparietal/default-mode networks, overweight individuals showed higher functional connectivity in visual, transmodal, and subcortical networks, whereas dorsal attention and somatomotor networks showed weaker connectivity. Associating with eating behaviors, visual and somatomotor networks showed negative correlations with hunger and disinhibition-related behaviors. Our findings provided insights into how functional hierarchical organization is disrupted in overweight individuals and suggest potential links between our results and eating behaviors.

SFC analysis identifies seed-based connectivity patterns at different step distances and this approach efficiently examines how brain systems reconfigure their modes of operation along the axis of brain hierarchy^27^. A previous study introduced SFC analysis in neuroimaging and found a dynamic transition of functional connectivity along the hierarchical axis ranging from primary sensory to higher-order cognitive control networks, which are represented as cortical hubs^27^. In healthy controls, SFC patterns show a clear cortical hierarchy in which sensory information converges to higher-order heteromodal association areas^27,30^. The SFC has been applied to compare age-related differences in hierarchical structures in infants^28^, and to assess disease-related connectome perturbations^29–31^. Indeed, individuals with autism spectrum condition showed that sensory-driven connectivity did not converge to default-mode regions, and this alteration was associated with disturbed social cognition and repeated behavioral symptoms^30^. Other studies have reported atypical convergence patterns of SFC in attention-deficit/hyperactivity disorder, especially within sensory and between sensory and cognitive control regions^29,31^. These studies collectively show the effectiveness of SFC analysis for capturing typical and atypical hierarchical organization of healthy and diseased populations. Hierarchical organization of the brain also exists in individuals with obesity. Our SFC analysis led to differential transition patterns of functional connectivity in multiple networks, including lower-level sensory and higher-order limbic networks, suggesting a disrupted organization of the functional hierarchy in individuals with obesity. These disrupted patterns were consistent when we changed the seeds to cognition, reward, and sensorimotor-related regions, indicating the robustness of SFC analysis irrespective of the seeds.

In addition to the SFC at different steps, we performed a correlation analysis between functional degree and eating behavior traits to provide behavioral underpinnings of our findings. The results of our study indicate that increases in functional connectivity in sensorimotor networks are associated with decreased hunger and disinhibited traits. Our work confirms prior work in individuals with eating disorders, where patients with anorexia display increased brain activation in somatosensory regions, indicating a failure in sensory processing related to altered eating habits^37^. Functional anomalies in sensory networks may involve excitation of neuronal cells in the basal forebrain because appetite suppression and food avoidance are known to be regulated by excitatory basal forebrain circuits that integrate external sensory information^38^. Previous studies suggested the association across brain function, dimensions of eating behavior, and body weight^39–42^. For example, eating behaviors were shown to mediate genetic susceptibility to obesity^43^. The fat mass and obesity-associated (FTO) gene is a key allele of obesity moderating satiety responsiveness, food intake, and binge eating, and the variational expressions in FTO lead to obesity^44^. In studies using structural MRI, abnormal FTO expressions were associated with decreases in gray matter volume in frontal and occipital regions in individuals with higher BMI^45–49^. Variations in the FTO gene were also associated with sensitivity of brain activity in reward and impulse/inhibitory control of eating, predisposing to develop obesity^50–52^. Further validation to understand the underlying neuronal mechanisms of sensory-related functional alterations in overweight individuals is still needed. In addition, while we found a significant association between functional connectivity in visual network and TFEQ-hunger subscale, it should be noted that the hunger subscale is relatively unstable across different populations, and thus needs to be carefully interpreted^53^.

This study had several limitations. First, we analyzed the data obtained from a single cohort owing to the limited information on eating behavior scores in other databases. Further validation using independent data can be performed in future studies. Second, we assessed step-related differences in functional connectivity only. Structural connectivity based on diffusion tractography or multimodal integration, such as structure-function coupling, may provide additional insights into obesity phenotype-related connectome perturbations. Third, although we excluded subjects with medical conditions or related medications, we could not control obesity-related medical complications, such as metabolic syndromes and hypertension, as well as physiological effects, which may affect brain function. Future studies should collect such information and replicate the findings to assess their robustness. Fourth, prior works investigated associations between longitudinal changes in BMI and functional connectivity in sensorimotor and frontoparietal networks^54,55^. However, how the whole-brain-wide functional hierarchy changes are related to weight gain or loss need to be explored further using longitudinal study designs. Lastly, although many studies adopted TFEQ to assess physical and psychological traits in individuals with obesity, it is a subject measurement based on self-report questionnaires^22,41,56,57^. In future works, more objective tools need to be developed.

In this study, we investigated differences in whole-brain functional connectivity profiles between individuals with healthy weight and overweight based on SFC analysis. We observed marked differences in brain regions along the functional hierarchical axis. In particular, sensorimotor networks showed significant associations with eating behaviors. In summary, our findings provide insights into the whole-brain-wide functional connectome organization in overweight individuals and their behavioral expression related to eating behaviors.

## Methods

### Participants and imaging data

This retrospective study was approved by the Institutional Review Board (IRB) of Sungkyunkwan University and was performed in full accordance with the local IRB guidelines. All participants provided informed consent. We obtained T1-weighted structural MRI and rs-fMRI data from the enhanced Nathan Kline Institute-Rockland Sample (eNKI) database^32^. Among 650 participants, subjects with medical conditions (e.g., attention-deficit/hyperactivity disorder, depression, migraine, diabetes, and cardiovascular diseases) or related medication (*n* = 165), and lack of full demographic information and obesity phenotypes (BMI and WHR; *n* = 184) were excluded. A total of 301 participants were included in this study. Detailed demographic information is reported in Table 2, and the distribution of obesity phenotypes of all participants is represented in Supplementary Fig. 11.

**Table 2:** Demographic information of study participants of healthy weight (18.5 ≤ BMI < 25 and WHR ≤ 0.85/0.9 for female/male) and overweight (BMI ≥ 25 and WHR > 0.85/0.9) individuals.

Information		Healthy weight	Overweight	*p*-value
Number of participants		104	75	N/A
Age		35.87 ± 17.32	49.46 ± 17.22	5.63 × 10^−7^*
Sex (male: female)		37: 67	41: 34	1.10 × 10^−2^*
BMI (kg/m^2^)		22.37 ± 1.67	31.07 ± 4.60	4.07 × 10^−41^*
WHR	Male	0.81 ± 0.03	0.98 ± 0.06	1.12 × 10^−24^*
	Female	0.76 ± 0.05	0.90 ± 0.04	1.45 × 10^−25^*
TFEQ scores	Total	14.71 ± 7.05	18.83 ± 8.57	5.52 × 10^−4^*
	Dietary restraint	6.61 ± 4.62	8.99 ± 4.81	1.00 × 10^−3^*
	Disinhibition	3.79 ± 2.53	5.52 ± 3.31	1.06 × 10^−4^*
	Hunger	4.32 ± 3.21	4.32 ± 3.24	9.96 × 10^−1^

*BMI* body mass index, *TFEQ* three-factor eating questionnaire.

Mean ± standard deviation are reported if applicable. We reported waist-to-hip ratio (WHR) for males and females separately as the cut-off for distinguishing healthy weight and overweight groups are different between biological sexes. Information that showed significant between-group differences based on two-sample *t* test (for continuous variables) or chi-squared test (for discrete variables) are reported with asterisks.

All imaging data were scanned using a 3-T Siemens Magnetom Trio Tim scanner. The T1-weighted structural data were scanned using magnetization-prepared rapid gradient-echo (MPRAGE) sequence (repetition time [TR] = 1900 ms, echo time [TE] = 2.52 ms, flip angle = 9°, field-of-view [FOV] = 250 mm × 250 mm, 1 mm^3^ voxel resolution, and 176 slices). The rs-fMRI parameters were scanned using a multiband echo planar imaging (EPI) sequence (TR = 645 ms, TE = 30 ms, flip angle = 60°, FOV = 222 mm × 222 mm, 3 mm^3^ voxel resolution, 40 slices, and 900 volumes).

### Data preprocessing

All imaging data were preprocessed using a Fusion of Neuroimaging Preprocessing (FuNP) volume-based pipeline (https://gitlab.com/by9433/funp), which integrates AFNI, FSL, and ANTs software^58–61^ (Fig. 1a). The T1-weighted structural data were de-obliqued and reoriented in the right-posterior-inferior direction. The magnetic field inhomogeneity was corrected, and the nonbrain tissues were removed. The rs-fMRI data were preprocessed as follows: volumes of the first 10 s were discarded, the head motion was corrected, and intensity was normalized across the 4D volumes. Nuisance components of head motion, cerebrospinal fluid, white matter, and cardiac- and large-vein-related artifacts were regressed out using the FMRIB’s ICA-based Xnoiseifier (FIX)^62^. The cleaned rs-fMRI data were registered onto the T1-weighted data and subsequently onto the 3 mm isotropic Montreal Neurological Institute (MNI) standard space. Spatial smoothing with a full width at a half maximum of 5 mm was applied.

### Definition of seed regions for stepwise functional connectivity

We constructed functional connectivity matrix from the preprocessed rs-fMRI data using partial correlation with L2-norm (ridge regularization) of time series between different brain regions^63–65^ defined using the Brainnetome atlas^34^ (Fig. 1b). We set the regularization parameter with 0.5 derived from an existing study^66^. SFC analysis requires the seed regions to be specified. Following our recent study^21^, we opted for degree centrality, a graph-theoretical measure assessing the total strength of connections of a given region, to associate it with an obesity phenotype (i.e., WHR), which is a better factor predicting obesity-related complications compared to BMI^67–69^. We linearly correlated degree centrality with WHR for every region and corrected multiple comparisons using the false discovery rate (FDR) (*q* < 0.05)^70^. The brain regions that showed significant associations were selected as seeds for further SFC analysis. To visualize the associations at a large-scale network level, we stratified the correlation coefficients of the identified regions based on seven functional communities^33^, as well as subcortical regions of amygdala, hippocampus, globus pallidus, nucleus accumbens, putamen, caudate, and thalamus^71^. To provide robustness of our findings, we conducted bootstrap-based assessments 1000 times. We randomly selected 90% of participants with replacement and performed seed region identification by associating WHR and degree centrality values. We calculated linear correlations between the whole-brain-wide obesity phenotype-related map using the whole subjects with that using bootstrap samples. For each iteration, we conducted the SFC analysis. We additionally calculated a linear correlation between BMI and degree centrality to assess whether different obesity phenotypes (i.e., BMI and WHR) show consistent results.

### Stepwise functional connectivity analysis

Seeding from the seed regions, we applied SFC analysis to the 95% thresholded and binarized connectivity matrix for each individual (Fig. 1c). We then assessed how the whole-brain functional connectivity changes their organization at different step distances^27^. Specifically, we counted the number of all paths that connect a seed region and target regions (i.e., whole brain) at a given step distance^27^. For each step, the SFC matrix was *z*-normalized. We assigned each participant to one of two distinct groups of healthy weight or overweight based on BMI and WHR (healthy weight [*n* = 104]: 18.5 ≤ BMI < 25 and WHR ≤ 0.85 for female 0.9 for male; overweight [*n* = 75]: BMI ≥ 25 and WHR > 0.85 for female, 0.9 for male). For each step, we averaged individual SFC matrices within each group and compared the averaged matrices between the groups using two-sample *t* tests at a regional level. To further assess network-level differences, we averaged the degree centrality of the regions involved in the same brain network^33,71^, and computed between-group differences. Multiple comparisons were corrected for both tests using FDR (*q* < 0.05)^70^. Additional SFC analyses using seeds from several subnetworks were performed, where each subnetwork involved regions defined based on behavioral domains of the Brainnetome atlas (http://atlas.brainnetome.org/bnatlas.html).

### Association with eating behaviors

We explored the possible underlying behavioral traits of our findings. The degree centrality of the networks that showed significant between-group differences at the largest step distance was correlated with eating behavior traits measured by a three-factor eating questionnaire (TFEQ)^72,73^. The TFEQ included three subscales of dietary restraint, disinhibition, and hunger, as well as the total score. We correlated degree centrality values and each of the TFEQ scores based on permutation tests. Specifically, we randomly shuffled participants and correlated degree centrality values with each TFEQ score 5000 times. This process yielded a null distribution of correlation coefficients, and we considered the real correlation coefficient significant if it exceeded 95% of the distribution. We further corrected multiple comparisons across different TFEQ scores as well as brain networks using FDR^70^.

### Sensitivity analysis

- Age and sex. As age and sex showed significant differences between the individuals with healthy weight and overweight, we additionally performed SFC analysis after controlling these factors from the functional connectivity.
- TFEQ scores. As the eating behavior scores, especially dietary restraint and disinhibition, were significantly different between the groups, we conducted SFC analysis after controlling for TFEQ scores.
- Low- vs. high-health risk groups comparison. We investigated between-group differences in SFC between low- and high-health risk groups defined based on the world health organization (WHO) criteria^36^. The low-risk group (*n* = 184) had WHR < 0.80/0.95 for female/male, and high-risk group (*n* = 51) had WHR > 0.86/1.0. To adjust the imbalance of the number of participants between the groups, we implemented bootstrap-based assessment by randomly selecting 51 participants from the low-risk group, and compared the degree centrality values across the steps. We repeated the process 1,000 times.

### Statistics and Reproducibility

We computed Pearson’s correlation between degree centrality values of 246 brain regions and WHR to identify regions associated with obesity. Multiple comparisons across brain regions were corrected using Benjamini–Hochberg FDR procedure^70^. In SFC analysis, we performed two-sample *t* tests at a regional- and network-level to identify between-group differences in SFC pattern between healthy weight and overweight groups. We furthermore correlated the degree centrality values of the networks with eating behavior traits. We randomly shuffled participants and calculated Pearson’s correlation 5,000 times to build a null distribution. The *p*-value was determined by counting the number of null correlation coefficients larger or smaller than the real correlation coefficient (two-sided test). The robustness of the SFC patterns was assessed via a bootstrap-based approach, which randomly selected 90% of participants with replacement 1,000 times. We furthermore assessed the robustness by controlling for age, sex, and TFEQ scores that showed significant between-group differences using a linear regression model.

### Reporting summary

Further information on research design is available in the Nature Research Reporting Summary linked to this article.

## Supplementary information

Supplementary Information

Description of Additional Supplementary Files

Supplementary Data 1

Reporting Summary

## Data Availability

Data used in this study are available from the enhanced Nathan Kline Institute-Rockland Sample (eNKI-RS) database (https://fcon_1000.projects.nitrc.org/indi/enhanced/access.html). The eNKI-RS Institutional Data Access Committee grants access to researchers who meet the criteria for access to confidential data upon completion of the Data Usage Agreement. Researchers should contact the database administrator to get access to data. Source data are provided with this paper as Supplementary Data 1.

## Code availability

The codes for data preprocessing are available at https://gitlab.com/by9433/funp, for stepwise functional connectivity are at https://sites.google.com/site/bctnet, and for full analysis are at https://github.com/hebinalee/SFC_obesity.
