# Supplementary material for: Disrupted stepwise functional brain organization in overweight individuals
Source: Commun Biol. 2022 Jan 10;5:11. doi: 10.1038/s42003-021-02957-7 (PMC8748821; doi:10.1038/s42003-021-02957-7)
Supplement: Supplementary file 1 — Supplementary Information [file 42003_2021_2957_MOESM1_ESM.pdf]

Supplementary Information

Disrupted stepwise functional brain organization in overweight individuals

Hyebin Lee[1,2], Junmo Kwon[1,2], Jong-eun Lee[1,2], Bo-yong Park[2,3*], and Hyunjin Park[2,4*]

[1] Department of Electrical and Computer Engineering, Sungkyunkwan University, Suwon, Korea

[2] Center for Neuroscience Imaging Research, Institute for Basic Science, Suwon, Korea

[3] Department of Data Science, Inha University, Incheon, Korea

[4] School of Electronic and Electrical Engineering, Sungkyunkwan University, Suwon, Korea

*Corresponding authors:

E-mail: boyong.park@inha.ac.kr (BP), hyunjinp@skku.edu (HP)

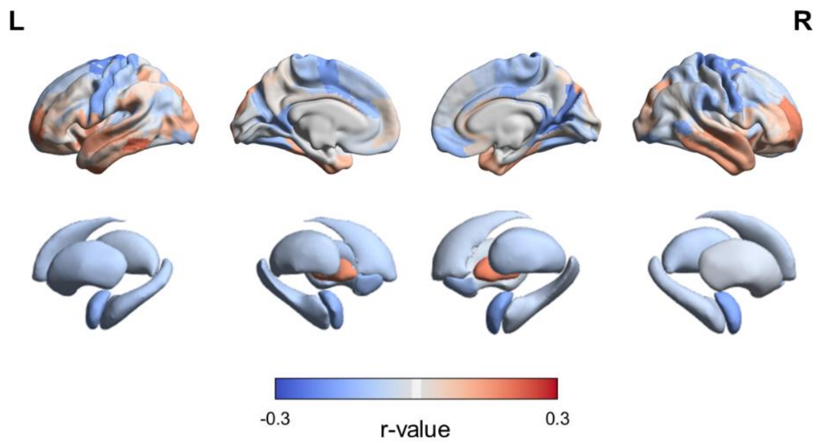

Supplementary Figure 1. Consistency of brain regions associated with obesity phenotype. We averaged correlation coefficients calculated based on the linear correlation between waist-to-hip ratio (WHR) and degree centrality values across 1,000 bootstraps and reported them on brain surfaces. *Abbreviations:* L, left; R, right.

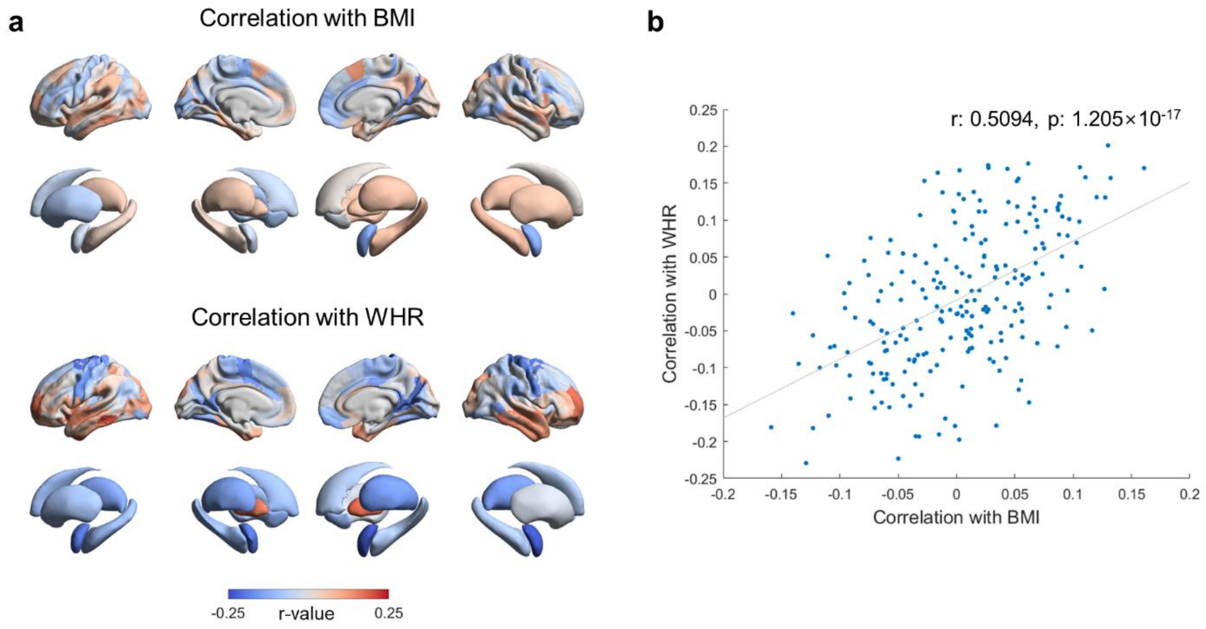

Supplementary Figure 2. Correlations between degree centrality and obesity phenotypes. a The correlation coefficients between degree centrality and obesity phenotypes of body mass index (BMI; top) and waist-to-hip ratio (WHR; bottom) are plotted on brain surfaces. **b** Linear correlation of the effects between BMI and WHR.

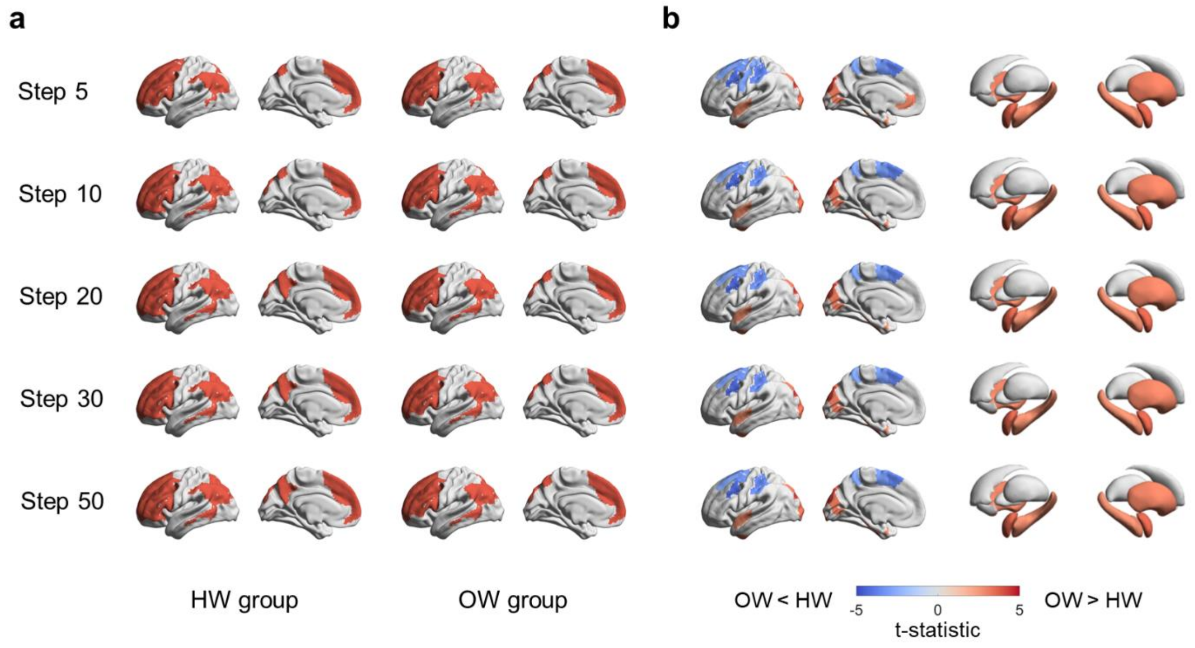

Supplementary Figure 3. Stepwise functional connectivity for higher step distances. a Hub regions across different step distances above step five, and **b** the t-statistics of brain regions that showed significant between-group differences in degree centrality. For details, see *Fig. 3*.

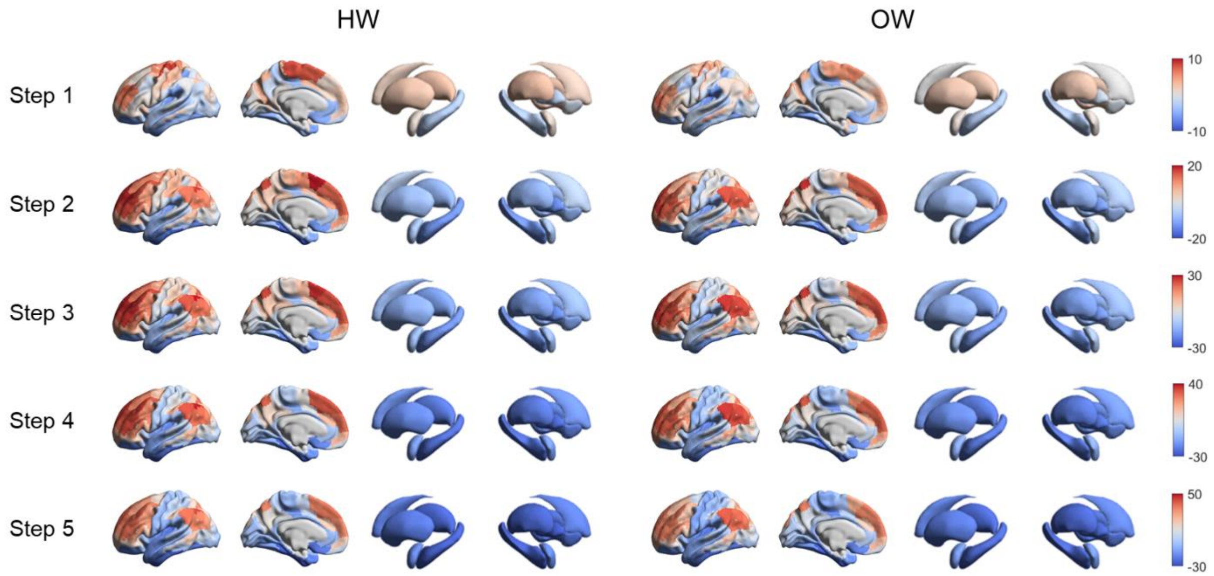

Supplementary Figure 4. Consistency of stepwise functional connectivity in healthy weight (HW) and overweight (OW) groups. We reported average degree centrality values for 1,000 bootstraps across different step distances.

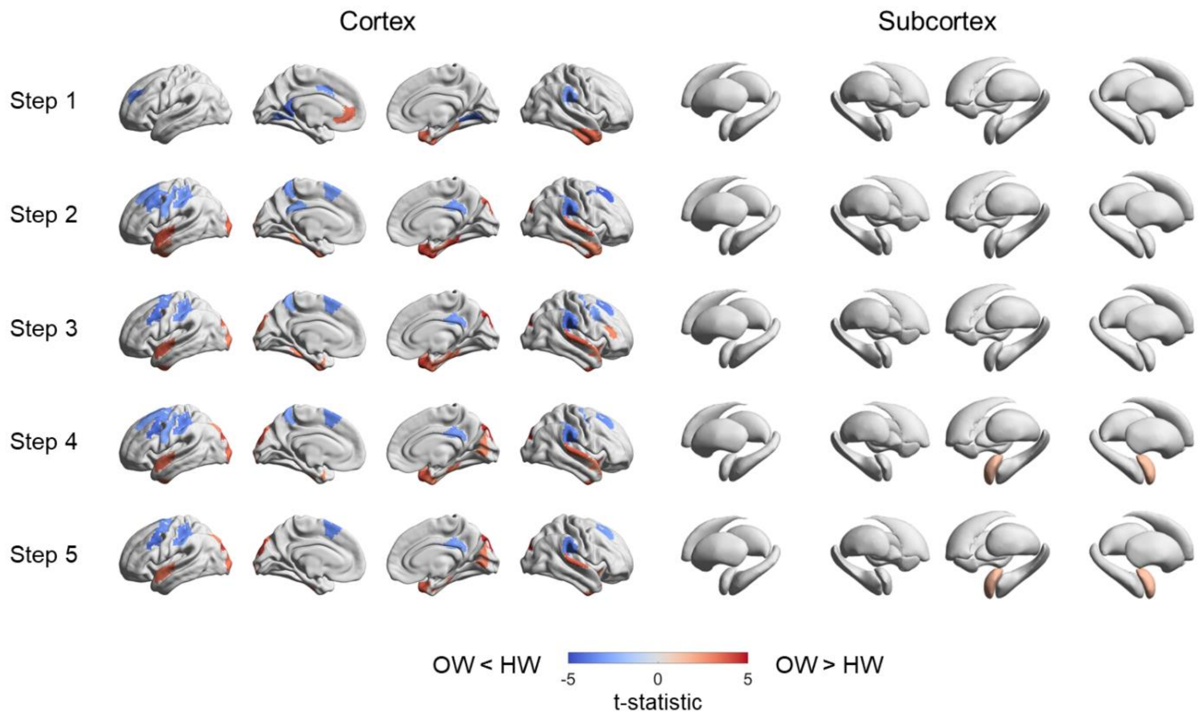

Supplementary Figure 5. Comparison of SFC degree pattern between healthy weight (HW) and overweight (OW) groups for the cognition-related seed regions. The regions that showed significant group differences in terms of degree centrality values are shown on brain surfaces and subcortex structures. For details, see *Fig. 3*.

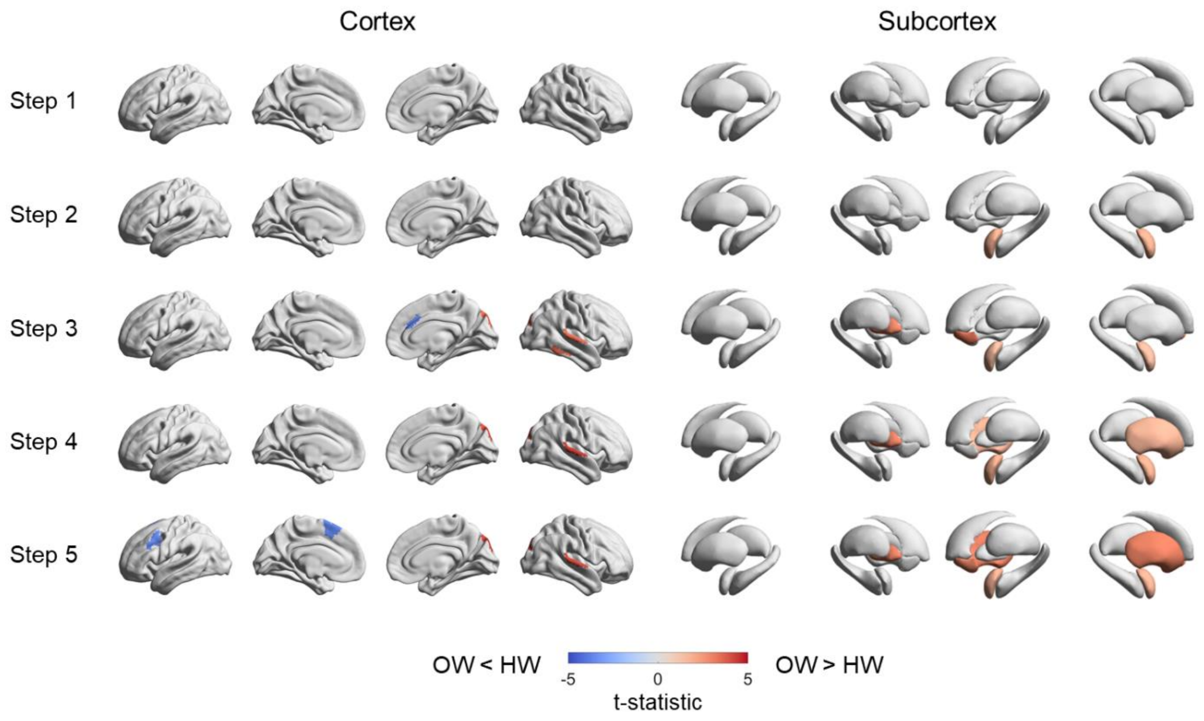

Supplementary Figure 6. Comparison of SFC degree pattern between healthy weight (HW) and overweight (OW) groups for the reward-related seed regions. The regions that showed significant group differences in terms of degree centrality values are shown on brain surfaces and subcortex structures. For details, see *Fig. 3*.

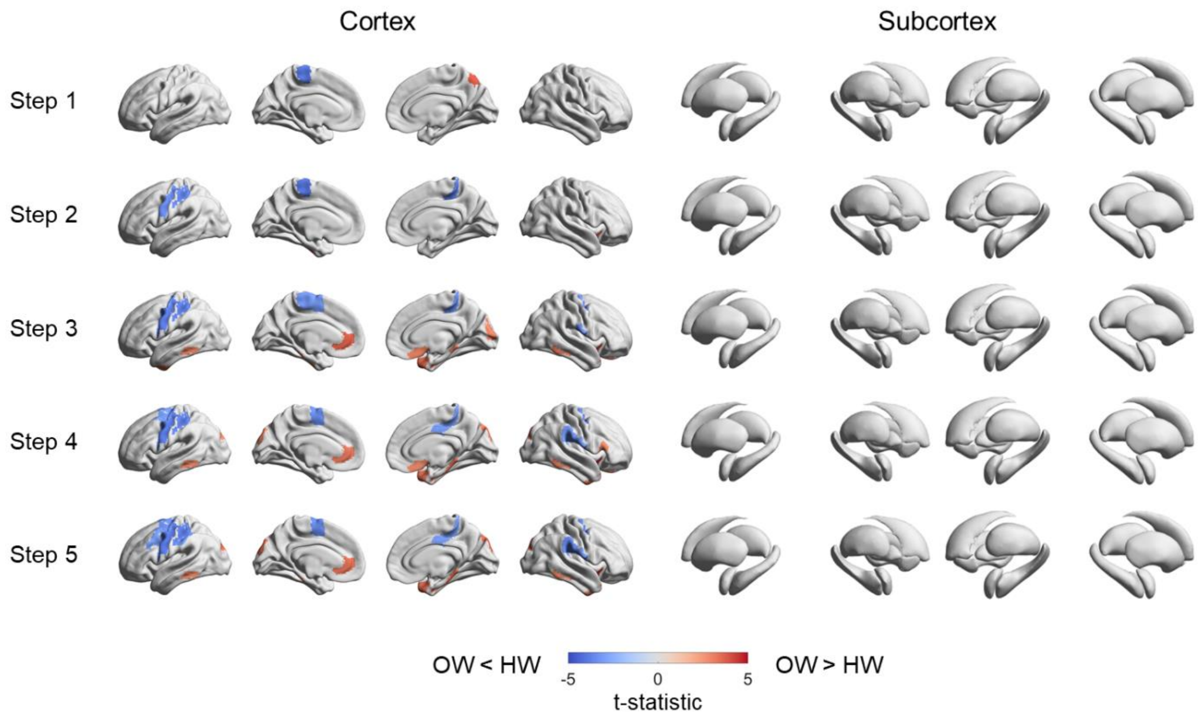

Supplementary Figure 7. Comparison of SFC degree pattern between healthy weight (HW) and overweight (OW) groups for the sensorimotor-related seed regions. The regions that showed significant group differences in terms of degree centrality values are shown on brain surfaces and subcortex structures. For details, see *Fig. 3*.

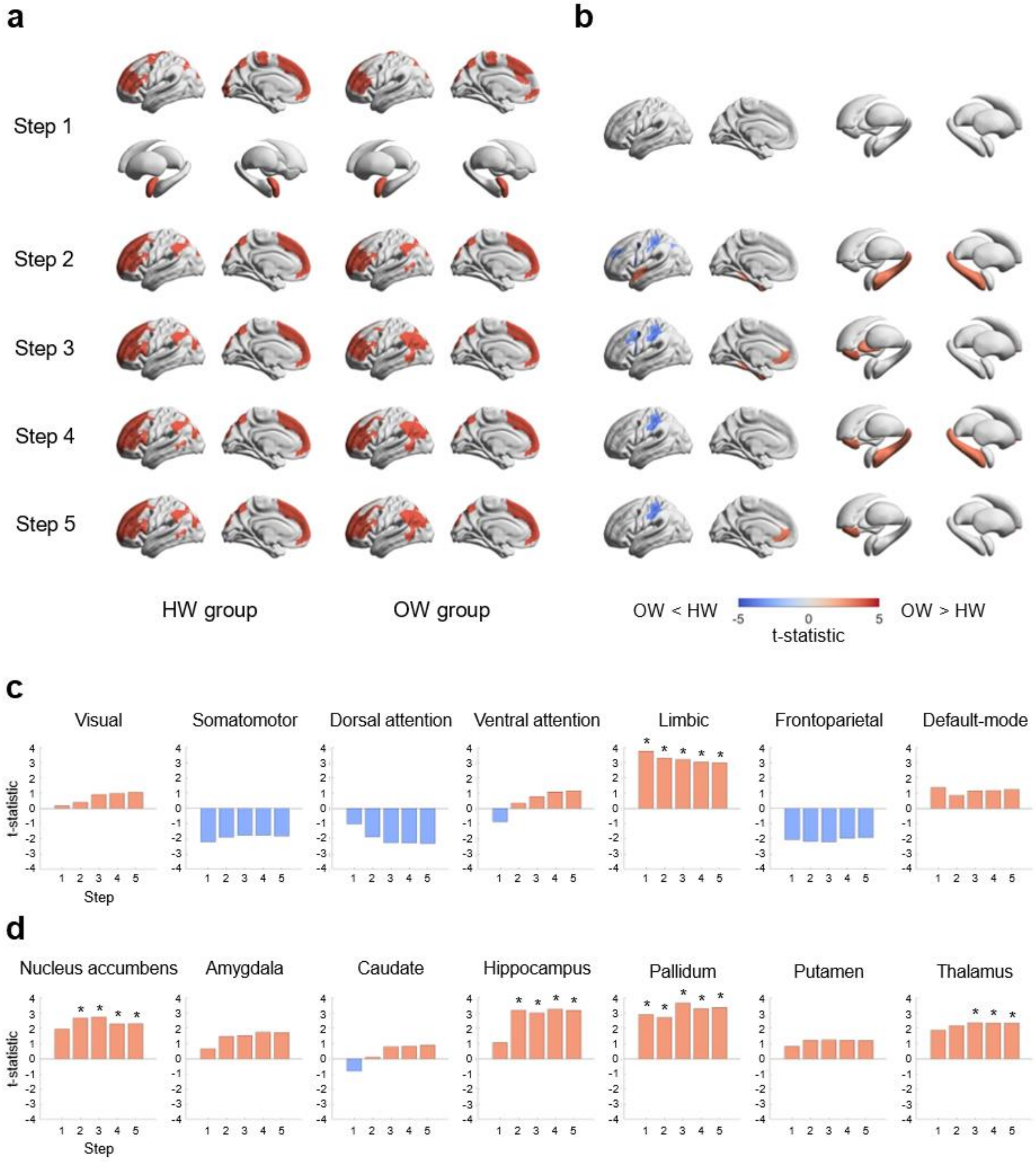

Supplementary Figure 8. Stepwise functional connectivity in healthy weight (HW) and overweight (OW) groups after controlling for age and sex. a Hub regions across different step distances, and **b** the t-statistics of brain regions that showed significant between-group differences in degree centrality. **c** Network-level and **d** subcortex-wise stratification of between-group differences. For details, see *Fig. 3* and *4*.

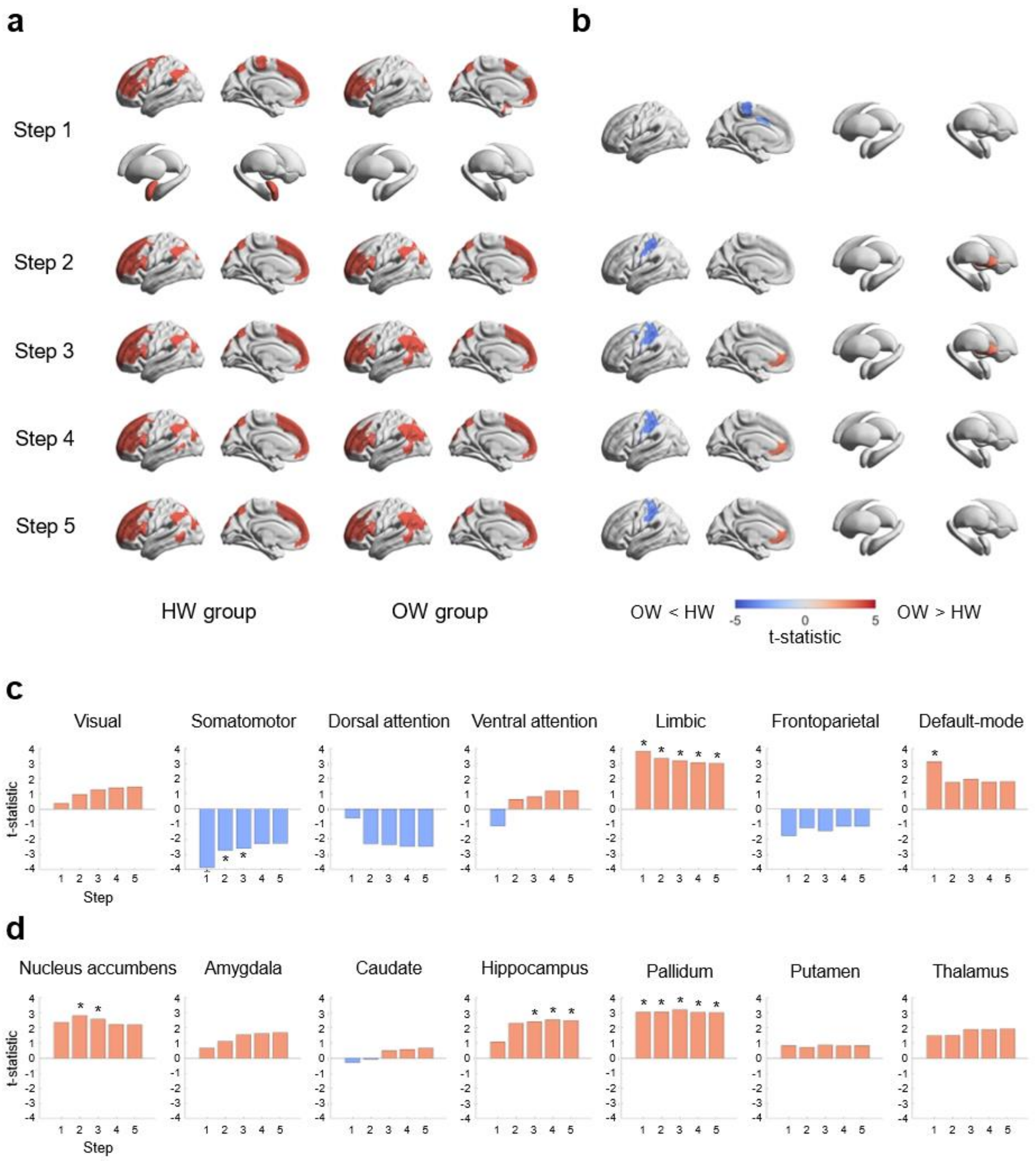

Supplementary Figure 9. Stepwise functional connectivity in healthy weight (HW) and overweight (OW) groups after controlling for eating behavior scores. a Hub regions across different step distances, and **b** the t-statistics of brain regions that showed significant between-group differences in degree centrality. **c** Network-level and **d** subcortex-wise stratification of the between-group differences. For details, see *Fig. 3* and *4*.

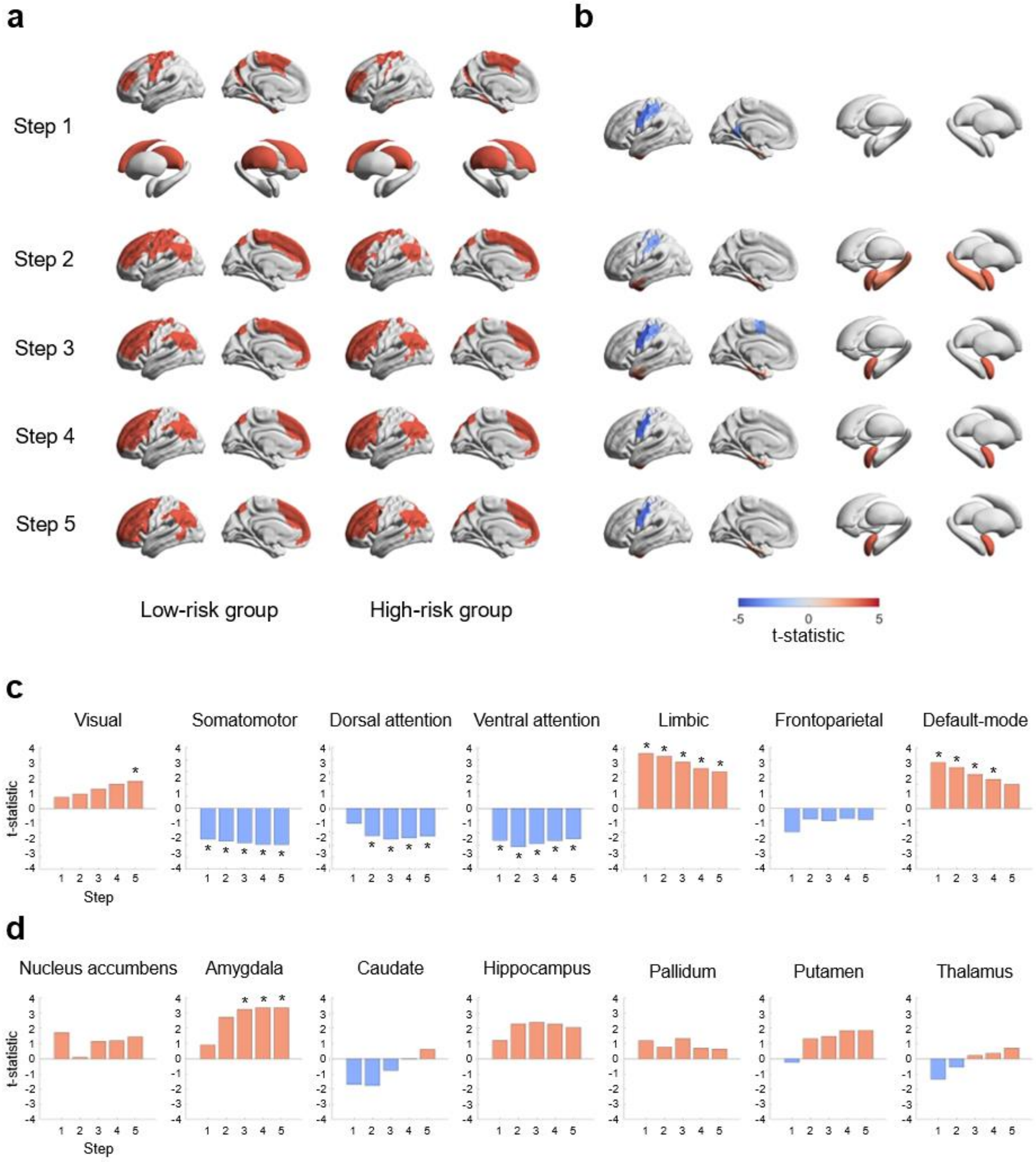

Supplementary Figure 10. Stepwise functional connectivity in low- and high-health risk groups. a Hub regions across different step distances, and **b** the t-statistics of brain regions that showed significant between-group differences in degree centrality. **c** Network-level and **d** subcortex-wise stratification of between-group differences. A representative iteration among 1,000 bootstraps was reported. For details, see *Fig. 3* and *4*.

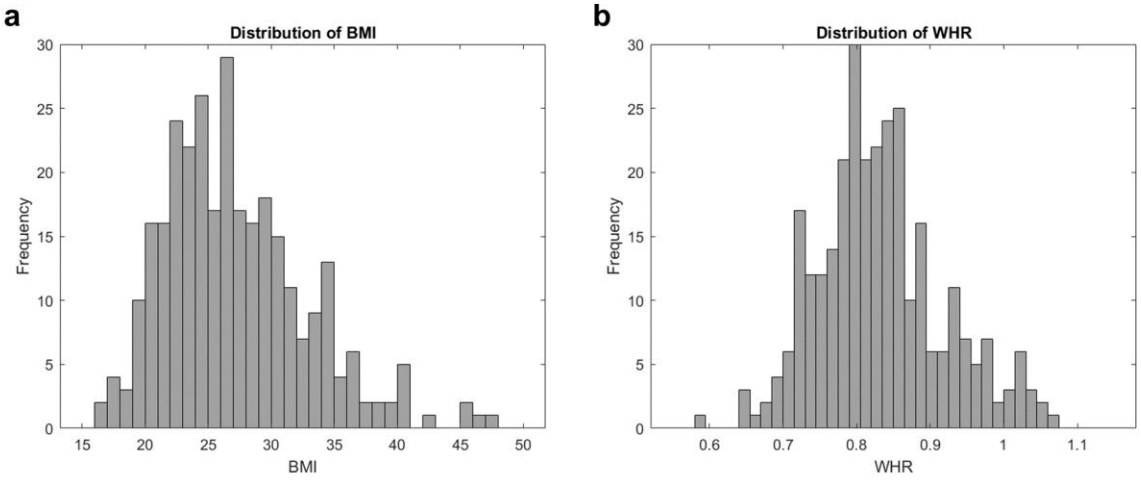

Supplementary Figure 11. Obesity phenotype distribution. a Distribution of body mass index (BMI) and **b** waist-to-hip ratio (WHR).
