# Supplementary material for: Disrupted stepwise functional brain organization in overweight individuals
Source: Commun Biol. 2022 Jan 10;5:11. doi: 10.1038/s42003-021-02957-7 (PMC8748821; doi:10.1038/s42003-021-02957-7)
Supplement: Supplementary file 2 — Description of Additional Supplementary Files [file 42003_2021_2957_MOESM2_ESM.pdf]

Description of Additional Supplementary Data

File name: Supplementary Data 1

Description: Source data files of main figures.
