# Supplementary material for: Disrupted stepwise functional brain organization in overweight individuals
Source: Commun Biol. 2022 Jan 10;5:11. doi: 10.1038/s42003-021-02957-7 (PMC8748821; doi:10.1038/s42003-021-02957-7)
Supplement: Supplementary file 4 — Reporting Summary [file 42003_2021_2957_MOESM4_ESM.pdf]

nature portfolio

Corresponding author(s): DBPR, COMMSBIO-21-1584A

Last updated by author(s): Nov 18, 2021

Reporting Summary

Nature Portfolio wishes to improve the reproducibility of the work that we publish. This form provides structure for consistency and transparency in reporting. For further information on Nature Portfolio policies, see our Editorial Policies and the Editorial Policy Checklist.

Statistics

For all statistical analyses, confirm that the following items are present in the figure legend, table legend, main text, or Methods section.

n/a	Confirmed	
☐	☒	The exact sample size (*n*) for each experimental group/condition, given as a discrete number and unit of measurement
☒	☐	A statement on whether measurements were taken from distinct samples or whether the same sample was measured repeatedly
☐	☒	The statistical test(s) used AND whether they are one- or two-sided<br>*Only common tests should be described solely by name; describe more complex techniques in the Methods section.*
☐	☒	A description of all covariates tested
☐	☒	A description of any assumptions or corrections, such as tests of normality and adjustment for multiple comparisons
☐	☒	A full description of the statistical parameters including central tendency (e.g. means) or other basic estimates (e.g. regression coefficient) AND variation (e.g. standard deviation) or associated estimates of uncertainty (e.g. confidence intervals)
☐	☒	For null hypothesis testing, the test statistic (e.g. *F*, *t*, *r*) with confidence intervals, effect sizes, degrees of freedom and *P* value noted<br>*Give P values as exact values whenever suitable.*
☒	☐	For Bayesian analysis, information on the choice of priors and Markov chain Monte Carlo settings
☒	☐	For hierarchical and complex designs, identification of the appropriate level for tests and full reporting of outcomes
☐	☒	Estimates of effect sizes (e.g. Cohen's *d*, Pearson's *r*), indicating how they were calculated

Our web collection on statistics for biologists contains articles on many of the points above.

Software and code

Policy information about availability of computer code

Data collection	We obtained T1-weighted structural MRI and rs-fMRI data from the enhanced Nathan Kline Institute-Rockland Sample (eNKI) database (http://fcon_1000.projects.nitrc.org/indi/enhanced/).
Data analysis	Data preprocessing: https://gitlab.com/by9433/funp<br>Stepwise functional connectivity: https://sites.google.com/site/bctnet<br>Full analysis: https://github.com/hebinalee/SFC_obesity

For manuscripts utilizing custom algorithms or software that are central to the research but not yet described in published literature, software must be made available to editors and reviewers. We strongly encourage code deposition in a community repository (e.g. GitHub). See the Nature Portfolio guidelines for submitting code & software for further information.

Data

Policy information about availability of data

All manuscripts must include a data availability statement. This statement should provide the following information, where applicable:

- Accession codes, unique identifiers, or web links for publicly available datasets
- A description of any restrictions on data availability
- For clinical datasets or third party data, please ensure that the statement adheres to our policy

Data used in this study are available from the enhanced Nathan Kline Institute-Rockland Sample (eNKI-RS) database (http://fcon_1000.projects.nitrc.org/indi/enhanced/access.html). The eNKI-RS Institutional Data Access Committee grants access to researchers who meet the criteria for access to confidential data upon completion of the Data Usage Agreement. Researchers should contact the database administrator to get access to data.

Field-specific reporting

Please select the one below that is the best fit for your research. If you are not sure, read the appropriate sections before making your selection.

☒ Life sciences ☐ Behavioural & social sciences ☐ Ecological, evolutionary & environmental sciences

For a reference copy of the document with all sections, see nature.com/documents/nr-reporting-summary-flat.pdf

Life sciences study design

All studies must disclose on these points even when the disclosure is negative.

Sample size	301 neurologically healthy controls
Data exclusions	Among 650 participants, subjects with medical conditions (e.g., attention-deficit/hyperactivity disorder, depression, migraine, diabetes, and cardiovascular diseases) or related medication (n = 165), and lack of full demographic information and obesity phenotypes (BMI and the waist-to-hip ratio [WHR]; n = 184) were excluded.
Replication	N/A
Randomization	Correlation analyses were performed using 5,000 permutation tests by randomly shuffling the participants. A null distribution was constructed, and if the real correlation coefficient did not belong to 95% of the distribution, we considered the correlation to be significant (two-tailed p < 0.05).
Blinding	N/A

Reporting for specific materials, systems and methods

We require information from authors about some types of materials, experimental systems and methods used in many studies. Here, indicate whether each material, system or method listed is relevant to your study. If you are not sure if a list item applies to your research, read the appropriate section before selecting a response.

Materials & experimental systems

n/a	Involved in the study	
☒	☐	Antibodies
☒	☐	Eukaryotic cell lines
☒	☐	Palaeontology and archaeology
☒	☐	Animals and other organisms
☐	☒	Human research participants
☒	☐	Clinical data
☒	☐	Dual use research of concern

Methods

n/a	Involved in the study	
☒	☐	ChIP-seq
☒	☐	Flow cytometry
☐	☒	MRI-based neuroimaging

Human research participants

Policy information about studies involving human research participants

Population characteristics	Number of participants: 301<br>Age: mean (SD) = 40.44 (17.68) years<br>Sex (male : female): 119:182<br>BMI (kg/m2): mean (SD) = 27.18 (5.66)<br>WHR: Male: mean (SD) = 0.88 (0.09); Female: 0.80 (0.07)<br>Healthy weight (18.5 ≤ BMI < 25) : Overweight (25 ≤ BMI < 30) : Obesity (BMI ≥ 30) = 123 : 97 : 81
Recruitment	We obtained T1-weighted structural MRI and rs-fMRI data from the enhanced Nathan Kline Institute-Rockland Sample (eNKI) database.
Ethics oversight	This retrospective study was approved by the Institutional Review Board (IRB) of Sungkyunkwan University and was performed in full accordance with the local IRB guidelines. All participants provided informed consent.

Note that full information on the approval of the study protocol must also be provided in the manuscript.

Magnetic resonance imaging

Experimental design

Design type	Resting-state
Design specifications	Resting-state
Behavioral performance measures	Resting-state

Acquisition

Imaging type(s)	T1-weighted, resting-state fMRI
Field strength	3T
Sequence & imaging parameters	All imaging data were scanned using a 3-T Siemens Magnetom Trio Tim scanner. The T1-weighted structural data were scanned using magnetization-prepared rapid gradient-echo (MPRAGE) sequence (repetition time [TR] = 1900 ms, echo time [TE] = 2.52 ms, flip angle = 9°, field-of-view [FOV] = 250 mm × 250 mm, 1 mm3 voxel resolution, and 176 slices). The rs-fMRI parameters were scanned using a multiband echo planar imaging (EPI) sequence (TR = 645 ms, TE = 30 ms, flip angle = 60°, FOV = 222 mm × 222 mm, 3 mm3 voxel resolution, 40 slices, and 900 volumes).
Area of acquisition	whole brain

Diffusion MRI ☐ Used ☒ Not used

Preprocessing

Preprocessing software	All imaging data were preprocessed using a Fusion of Neuroimaging Preprocessing (FuNP) volume-based pipeline, which integrates AFNI, FSL, and ANTs software.
Normalization	The cleaned rs-fMRI data were registered onto the T1-weighted data and subsequently onto the 3 mm isotropic Montreal Neurological Institute (MNI) standard space.
Normalization template	3 mm isotropic Montreal Neurological Institute (MNI) standard space.
Noise and artifact removal	Nuisance components of head motion, cerebrospinal fluid, white matter, and cardiac- and large-vein-related artifacts were regressed out using the FMRIB's ICA-based Xnoiseifier (FIX).
Volume censoring	Head motion was corrected using FSL.

Statistical modeling & inference

Model type and settings	Linear correlation<br>Two-sample t-tests
Effect(s) tested	r-values and FDR-corrected p-values<br>t-statistics and FDR-corrected p-values

Specify type of analysis: ☐ Whole brain ☐ ROI-based ☒ Both

Anatomical location(s): Brainnetome atlas

Statistic type for inference (See Eklund et al. 2016)	ROI-wise
Correction	FDR

Models & analysis

n/a	Involved in the study
☐	☒ Functional and/or effective connectivity
☐	☒ Graph analysis
☒	☐ Multivariate modeling or predictive analysis

Functional and/or effective connectivity	Partial correlation with L2-norm (ridge regularization)
Graph analysis	Individual-level weighted graph<br>We opted for degree centrality, a graph-theoretical measure assessing the total strength of connections of a given region, to associate it with an obesity phenotype.
